# Effectiveness of a tinnitus management programme: a 2-year follow-up study

**DOI:** 10.1186/1472-6815-9-6

**Published:** 2009-06-26

**Authors:** Claire Gudex, Preben H Skellgaard, Torben West, Jan Sørensen

**Affiliations:** 1 CAST – Centre for Applied Health Services Research and Technology Assessment, University of Southern Denmark, Odense, Denmark; 2 Centre for Help Aids and Communication, Aabenraa, Denmark

## Abstract

**Background:**

Tinnitus impairs the possibility of leading a normal life in 0.5–1% of the population. While neither medical nor surgical treatment appears effective, counselling may offer some relief. An intervention combining counselling and hearing devices is offered to clients referred to the Centre for Help Aids and Communication (CHC) in southern Denmark. The aims of this exploratory study were to examine i) the characteristics of CHC's clients and their tinnitus, ii) the effectiveness of the treatment, and iii) whether particular client groups benefit more than others.

**Methods:**

One hundred new clients presenting with tinnitus completed the Tinnitus Handicap Inventory (THI) three times – before their first consultation, after one month and after 1–2 years. The scores were tested for significant differences over time using tests for paired data. Logistic regression was used to examine factors associated with a clinically important difference (i.e. THI score improvement of at least 20 points).

**Results:**

At final follow-up, total THI score was significantly lower than baseline, i.e. 29.8 (CI 25.5–34.2) vs. 37.2 (CI 33.1–37.2), p < 0.01. The programme achieved a clinically important difference for 27% and 24% of the clients one month and 1–2 years after the first consultation, respectively. It appeared that greater improvement in THI score was related to higher baseline THI score and possibly also to treatment by a particular CHC therapist. The absolute reduction in mean THI score after 1–2 years for clients with moderate and severe handicap was 14 and 20 points, respectively, i.e. similar to that previously reported for TRT (14–28 points). The cost of the current programme was approximately 200 EUR per client.

**Conclusion:**

The tinnitus management programme appeared to provide significant benefit to many clients at a relatively low cost. It would be useful to conduct a randomised controlled study comparing the current programme with alternative forms of combination counselling/sound therapy approaches.

## Background

The prevalence of tinnitus in the general population appears to be 8–15% [1-3] and it is suggested that 0.5–1% of the population has tinnitus to such a degree that their possibility of leading a normal life is affected [4-6]. While no effective medical or surgical treatment has yet been found for tinnitus [7-9], approaches that offer counselling may be more promising [7].

Tinnitus Retraining Therapy (TRT) [10] is an approach used in Denmark and elsewhere that combines counselling and sound therapy. Its goal is habituation of both the reaction to the tinnitus and the perception of the tinnitus signal itself. The main components are directive counselling and use of noise generators, and treatment typically continues over 1–2 years. While observational studies have reported significantly improved patient outcome after treatment with TRT [11,12], a review undertaken in 2000 of TRT studies [13] found no convincing evidence that TRT was superior to other treatments. Disadvantages of TRT have been reported as the long time period before the patient experiences benefit, high costs related to adaptation of the prosthesis, and high dependence on successful counselling [12]. This has lead to simplified forms of TRT being developed [14].

The treatment offered by the Centre for Help Aids and Communication (CHC), which serves the Danish county of Southern Jutland, is one of the alternatives to TRT in Denmark and comprises several key elements:

• Thorough patient history related to the development of tinnitus and previous treatment, ENT examination (in collaboration with local ear, nose and throat specialists)

• Full explanation about the mechanisms behind the development of tinnitus, possible causes, treatment strategies

• Individual advice and counselling about personal coping strategies, work-related problems

• Trials with various devices, e.g. hearing aids that strengthen high frequencies, and bedside maskers to deflect attention away from the tinnitus at night time. Tinnitus maskers (alone or in combination with hearing aid) are not used, as in CHC's experience clients do not use them.

By August 2005 CHC had used this treatment programme with 120 people presenting with tinnitus, and wished to quantify client outcome from the treatment. A randomised controlled study design was rejected due to the relatively small number of persons presenting to CHC with tinnitus, who were moreover a very mixed group with respect to cause, duration and severity of tinnitus. Instead, an exploratory study was undertaken, in the form of a prospective cohort study.

The aims of this exploratory study were to examine i) the characteristics of CHC's clients and their tinnitus, ii) the effectiveness of the treatment, and iii) whether particular client groups benefited more than others. A full report of the study and results is available (in Danish)[15].

## Method

### Cohort study

Potential study participants were all clients referred to CHC for treatment of tinnitus between 30th October 2005 and 30th October 2006. Exclusion criteria were age under 18 years, earlier treatment at CHC, non-Danish speakers and declining to participate in the study. All participants gave signed written consent for participation.

The intervention was CHC's standard treatment for tinnitus. Each client attended a first consultation with one of three CHC hearing therapists, in which the client's tinnitus experiences were discussed as a dialogue between therapist and client. Information collected included sociodemographic data, description of the tinnitus (acute/chronic start, duration, strength, location, sounds experienced, times of greatest disturbance), oversensitivity to noise, deafness, previous treatments, and general illness history and use of medicines. The therapist gave advice and counselling (especially regarding the nature of tinnitus, treatment possibilities and coping strategies), organised a trial of an open-fitted hearing aid (either Phonak or GN Resound devices were offered alternatively to consecutive clients) and/or bedside masker, clients were referred to a labour market consultant as necessary. Clients were invited for further consultations as necessary and all were welcome to telephone CHC or attend the open daily clinic in the event of questions or problems.

### Outcome measurement

Clients were asked to complete and bring to the first consultation a questionnaire that included the Tinnitus Handicap Inventory (THI). Two further postal questionnaires were completed one month and 12–22 months, respectively, after the first consultation. The follow-up questionnaires asked about changes in clients' tinnitus, lifestyle and work status, medicine use, alternative treatments sought and satisfaction with CHC's treatment. Resource use was estimated from an inspection of CHC's time registration for a small group of randomly selected clients.

The THI is a self-report measure of perceived tinnitus handicap [16,17] that can be used to grade the severity of tinnitus [5]. The instrument comprises 25 items, each of which has a 3-point response scale, i.e. Yes (4 points), Sometimes (2 points), No (0 points). Using the total score (0–100) tinnitus severity can be graded as none, mild, moderate and severe. The Danish version used in this study was previously validated among clients with severe continuous tinnitus [18,19]. The minimum sample size to allow identification of THI score differences was estimated to be 50 clients, based on 90% power, alpha of 0.05 and THI score standard deviation of 20 (from previous studies [11,12,20]).

Data were analysed using SPSS. Pre- and post-intervention scores were tested for significant differences using tests for paired data (Wilcoxon test for interval variables, sign test for ordinal variables, McNemar's test for dichotomous variables) and a significance level of p < 0.05.

Logistic regression analysis was used to investigate factors associated with a clinically important change in THI score; the THI developers suggest this to be at least 20 points [17]. The dependent variable in the regression analysis was change in THI score between baseline and 2^nd ^and 3^rd ^follow-up, respectively. Four types of variables were included as independent variables: *sociodemographic *(client age, gender, profession), *tinnitus *(duration, subjective loudness, type of sound), *burden *(baseline THI score, sound intolerance) and *treatment *(CHC therapist, type of hearing aid, client lifestyle changes).

## Results

### Client sample

Between 1^st ^November 2005 and 30^th ^October 2006, 130 clients were referred to CHC for the treatment of tinnitus. Four clients declined to participate in the study and of the rest, 122 and 102 completed the second and third questionnaires, respectively. There were 100 clients who completed all three questionnaires, for whom the results are presented here. There were no significant differences in sociodemographic or tinnitus variables between these 100 clients and the 126 clients who completed the first questionnaire (Table 1).

**Table 1:** Characteristics of participants at inclusion into the tinnitus study

	Completed first questionnaire (n = 126)	Completed all three questionnaires (n = 100)
% Men/women	67/33	65/35
Mean age (SD)	55 ± 11 yrs	56 ± 11 yrs
Range	(28–79)	(28–79)
Mean duration of tinnitus		
≤ 1 yr	19%	20%
2–10 yrs	42%	43%
>10 yrs	39%	36%
Likely aetiology		
Noise at work^a^	69%	64%
Stress	30%	31%
Sudden loud noise^b^	25%	23%
Cervical spine problems	14%	15%
Other^c^	41%	38%
Sound intolerance		
Client self-report	53%	54%
UCL 105–110 bilaterally	19%	14%
UCL 75–100	73%	77%
UCL ≤70 uni/bilaterally	8%	9%

^a ^Clients included machine workers, plane mechanics, truck drivers, teachers, musicians, bell ringer.

^b ^E.g. rifle fire, fireworks.

^c ^Included middle ear disease, whiplash, noise during leisure activities, back/neck/head injury.

Most clients (85%) were referrals from ENT specialists and 15% from the regional audiology clinic. Half were in active employment; of the 77 clients under 65 years, 33% were on sickness benefit or had taken early retirement. Noise at work was the likely cause of tinnitus for many clients (Table 1); 33% were employed in the industrial/tradesman sector. Tinnitus had developed gradually in 73% of clients and had been present for over 10 years in 36%. The description of the tinnitus varied, although high-frequency tones were the most common; 42% of clients experienced multiple tinnitus sounds. One-third of clients reported that tinnitus was a constant problem; the others experienced most problems when falling asleep or during the night. Most clients had evidence of sound intolerance (Table 1) and 70% had self-reported hearing loss in one or both ears. While only 5% used a hearing aid, many had tried a range of strategies, the most common being listening to music, going for a walk and other exercise, use of hearing earmuffs or ear plugs, but also acupuncture, chiropractor, massage and other forms of naturopathy.

### Intervention

A range of issues and strategies were discussed during the client consultations (Table 2), which had a mean duration of 107 ± 20 minutes (range 60–150 minutes). Recurring themes were lowering expectations to oneself, relaxation strategies, greater use of devices such as bedside maskers (which many clients already had at home) and custom-made hearing protectors. Nearly all clients (including some without self-reported hearing loss) were offered either a unilateral hearing aid or bilateral hearing aids to help mask the tinnitus.

**Table 2:** Main components of CHC's tinnitus management programme (n = 100 clients)

*CHC's intervention: advice and devices*	
Take more care of oneself/lower expectations to oneself	21%
Relaxation strategies	18%
Changes in work or general activity levels	13%
Trial of bedside masker/hearing protector	11%
Trial of hearing aid	94%
Other^1^	30%
*Clients' own interventions over 1–2 years:*	
Tried to be less stressed in daily life	48%
Tried to take more care of oneself	52%
A healthier lifestyle	49%
More stress-reducing activities	39%
Other^2^	9%

^1 ^E.g. radio as background noise, cognitive therapy, referral to general practitioner for further investigation, massage, chiropractor, alteration in mode of communication at home, greater use of the bedside masker that the client already had at home.

^2 ^Extra written comments from clients: e.g. avoidance of noise, use of hearing aid/hearing protector, weight loss, try to forget the tinnitus.

In the 1–2 years after the first consultation at CHC, all clients reported that they had made life-style changes to lessen the impact of the tinnitus (Table 2). The number of clients in active employment (n = 51) was more or less unchanged, but nine had altered their working hours or changed to another job. One-third of clients had contacted other health practitioners in connection with their tinnitus, including ENT specialist (8% of clients), physiotherapist (8%), acupuncturist (8%), chiropractor (3%) and others (5%, e.g. craniosacral therapy, zone therapy), while 8% reported activities such as relaxation and stress courses.

No major changes in medicines were reported. At baseline 40% of clients used no medicines at all and 13% took medicines that may have been related to tinnitus (tranquilisers, sleeping pills or natural medicine).

### Client outcome

At the time of the first consultation, the THI items most often experienced by clients were feeling a lack of control over the tinnitus (65% of clients), inability to escape from the tinnitus (55%), that stress made the tinnitus worse (48%), that the tinnitus influenced enjoyment of social activities (34%), and that the tinnitus made it difficult to fall asleep at night (32%). On the basis of total THI score, nearly 50% of clients had moderate or severe tinnitus (Table 3). At baseline, clients with subjectively louder tinnitus or sound intolerance had significantly higher (worse) THI scores; in the univariate analysis baseline THI score was not significantly related to other variables, including client age, gender, tinnitus duration or degree of self-reported hearing loss.

**Table 3:** THI scores over time for CHC clients with tinnitus (n = 100)

	Baseline		Follow-up after 1 month		Follow-up after 1–2 years	
	n	Mean score (95% CI)	n	Mean score (95% CI)	n	Mean score (95% CI)
Total THI score^1^	97	37.2 (33.1–37.2)	92	28.7 (24.6–32.7)**	92	29.8 (25.5–34.2)**
*Tinnitus handicap*^2^						
0–16 None	16	17%	32	35%*	30	33%#
18–34 Mild	35	36%	30	33%	31	34%
36–54 Moderate	30	31%	18	20%	18	20%
56–100 Severe	16	17%	12	13%	13	14%

^1^Total THI score is the sum of responses to the 25 items, where 'Yes' = 4 points, 'Sometimes' = 2 points and 'No' = 0 points. Total score ranges from 0–100; the higher the score, the greater the tinnitus handicap.

^2 ^Based on total THI score; from [12]

* Significantly better (p < 0.01) compared to baseline.

** Significantly better (p < 0.001) compared to baseline.

# p = 0.059

Clients' responses at follow-up showed statistically significant improvement on most THI items, e.g. fewer felt frustrated, irritable or desperate because of their tinnitus, fewer had problems concentrating or falling asleep at night and fewer felt that they could not escape their tinnitus. Significantly more clients felt they enjoyed life, had better control over the tinnitus and could cope better with it. Total THI score was significantly lower at both follow-ups, and fewer clients were categorised as having moderate or severe tinnitus handicap (Table 3). There were no significant differences between the responses to the 2nd and 3rd questionnaires.

Assuming that a 20-point difference represents a clinically important change on the THI, CHC's treatment gave a clinically important change for 27% and 24% of clients after one month and 1–2 years after the first consultation, respectively. Logistic regression (Table 4) showed that a 20-point improvement in THI score *one month after start of treatment *was associated with higher client age, shorter duration of tinnitus, higher baseline THI score and treatment by a particular CHC therapist. A 20-point improvement in THI score *1–2 years after start of treatment *was significantly associated with higher baseline THI score and (close to significance, p = 0.06) treatment by the same CHC therapist. While this CHC therapist had longer consultations than the other two (mean 116 min vs. 87 min, p < 0,001), he also treated 70% of the clients in the study, which may have resulted in an overestimation of his influence. His clients did not differ significantly from the others with respect to baseline THI score.

**Table 4:** Odds ratios estimated by logistic regression showing factors related to a 20-point improvement in THI score

	Follow-up questionnaire after 1 month		Follow-up questionnaire after 1–2 years	
	Odds ratio	p-value	Odds ratio	p-value
Age (continuous variable)	1,083	**0,012**	1,027	0,479
Woman (= 1) vs. Man	1,234	0,789	0,942	0,944
Skilled worker (= 1) vs. Others	1,447	0,637	0,793	0,782
Tinnitus duration: >5 years (= 1) vs. ≤ 5 years	0,205	**0,027**	1,130	0,869
Tinnitus strength: Loud or very loud (= 1) vs. Weaker	0,877	0,893	4,408	0,166
Tinnitus sounds: High-pitched whine/singing (= 1) vs. Others	0,520	0,415	0,478	0,448
Baseline THI score (continuous variable)	1,057	**0,005**	1,064	**0,007**
Sound intolerance: Yes (= 1) vs. No	2,718	0,193	0,656	0,599
CHC therapist: Therapist 1 (= 1) vs. Therapists 2+3	9,551	**0,016**	9,655	**0,062**
Hearing aid type: Phonak (= 1) vs. GN Resound	2,995	0,121	1,011	0,988
Client's lifestyle changes: Yes (= 1) vs. No	1,288	0,713	0,826	0,825
Constant term	0,000	0,000	0,001	0,012

There was a significant correlation between initial THI score and improvement in THI score. While the overall average improvement on THI was 8 points over 1–2 years, clients who had an initial THI score of 36–54 (moderate handicap) had an average improvement of 14 points, and those with initial score of 56 or over (severe handicap) had an average improvement of 20 points. The six clients who were not offered a hearing aid showed a slight worsening (by 2 points) in average THI score over time compared to the other clients who showed improvement (an average decrease of 9 points).

### Resource use

From an inspection of the time registration for a small group of randomly selected clients, the average time use per client was estimated to be just over four hours:

• Direct client contact: 3 hours and 10 minutes (consultations 2 hours, first adjustment of hearing aid 45 minutes, later adjustments 25 minutes; visits for hearing aid adjustments comprised both technical advice as well as counselling)

• Reporting in client journal, preparation prior to consultations, client contact after formal treatment completion: 55 minutes.

Assuming an average hourly payment rate to the therapist of €50, the treatment cost is approximately €200 per client, excluding contributions to fixed costs (overheads).

## Discussion

The results of this study indicate that CHC's clients with tinnitus derived significant benefit from the intervention. This benefit could already be observed one month after the first consultation and was at nearly the same level after 1–2 years. The improvements were emotional (e.g. less frustration, more enjoyment of life, greater control over tinnitus), cognitive (e.g. better concentration) and physical/social roles (e.g. fewer problems at work and at home, better sleep). It appeared that greater improvement in THI score was related to higher baseline THI score and possibly also to treatment by a particular CHC therapist.

The absolute mean reduction in THI score (8 points) was less than that reported for TRT (14–28 points) [11,12,21]. However, the average baseline score in the current study population was lower than that in the TRT studies. When the study clients were classified according to their initial THI score, those with moderate and severe handicap showed an average improvement of 14 and 20 points, respectively. A recent longitudinal study evaluating a simplified form of TRT reported a large average improvement of 45 points on the THI – although the mean baseline THI score was high (60 points) [14]. TRT uses directive counselling, involving a series of individualised educational sessions in which the patients are taught about the cause and mechanisms of tinnitus through demonstrations of anatomy and physiology; the use of neutral non-masking sound is also a central element of TRT. This intensive approach differs from the more traditional psychological counselling used at CHC, although the success of both approaches relies on building up a mutual rapport between client and counsellor. CHC's resource use has not been examined in detail in the current study, but with an average of four hours used per client (corresponding to a cost of approximately €200), the resource use is likely to be considerably less than that used in either a full TRT program or the simplified TRT form (typically seven sessions over 1–2 years [14]. This suggests that a randomised cost-effectiveness study comparing different (shorter) forms of combination counselling/sound therapy is warranted, including a more detailed cost analysis undertaken at individual client level.

Although CHC's treatment had helped the majority of clients, one-third of clients at follow-up were classified by their THI score as having moderate or severe handicap due to tinnitus. This finding is similar to that reported for patients five years after start of cognitive behavioural therapy [22]. Such a result is perhaps not surprising, given the long duration of tinnitus (nearly 40% of clients in the current trial had had tinnitus for at least ten years) and the major impact it had on their lives at baseline. Those who seek help for tinnitus may have more complex tinnitus sounds, higher degree of hearing loss and greater psychological effects than those who do not seek help [23,24]. In view of the nature of the intervention in the form of stress-reducing activities and changes in attitudes and self-expectations, improvement from treatment is likely to take some time. It is also possible, however, that some clients will not experience much improvement with CHC's treatment programme in its current form, and would benefit from other approaches.

### Limitations of the study

The pre-post study design cannot indicate how effective CHC's management programme is compared to other possible treatments. The lack of a control group means that one cannot be sure that the self-reported improvement with treatment was due to the effect of participating in a study or to clients feeling that they should answer positively after treatment. The intervention applied was no different to that the client would have received if the study had not been undertaken, however, and the written comments from the clients regarding their impression of and satisfaction from treatment (data not reported) suggest that they did feel free to report both positive and negative experiences.

Furthermore, only the results from clients with a full data set were included in the analysis. This meant that data from 20% of the clients who completed the first questionnaire were excluded. Although no significant differences in sociodemographic and tinnitus variables were found between these 26 clients and the 100 clients who completed all three questionnaires, there may have been other unidentified differences between the two groups, for example in attitude to tinnitus or response to the intervention.

The data showed that CHC's clients had very different needs and experiences with respect to their tinnitus. This was not only in relation to duration, sounds, strength and degree of disturbance from the tinnitus, but also regarding the treatment form and the time horizon for advice and lifestyle changes. It is possible that a more selected client group would produce different results. The relationship between audiological data and outcome measures was not analysed here, but would be relevant for future studies, e.g. outcome according to varying degree of hearing loss or type of audiogram.

Virtually all clients presenting to CHC in the course of a year participated in the study, and the 100 clients completing all three questionnaires were not significantly different on sociodemographic or clinical variables to the 126 with baseline data. The study population is thus probably representative of CHC's clients presenting with tinnitus. While the mean baseline score was lower than that in three TRT studies (two studies excluded patients with lower THI scores), it was similar to the baseline score in another Danish study [18].

Besides an assessment of how treatment influences clients' subjective evaluation of tinnitus and its effects on their daily life, it would be useful in a further study to enquire more deeply about clients' use of hearing aids, bedside maskers and other equipment. It was apparent at the start of the study that some clients had equipment at home that they did not use; it is not known how much this was due to uncertainty about how to work or use the equipment. In the current study there was no follow-up of the extent to which clients used the equipment provided by CHC; this would be useful information in assessing which aspect of the treatment was most effective.

## Conclusion

The tinnitus management programme appeared to provide significant benefit to many of its client at a relatively low cost. It would be useful to conduct a randomised controlled study comparing the current programme with alternative forms of combination counselling/sound therapy approaches. The heterogeneity of tinnitus clients with respect to cause, duration and severity of tinnitus, and presumably psychological and attitudinal differences, means that careful consideration would need to be given to sample size and selection and choice of control group.

## Competing interests

The study received equal financial support from GN Resound and Phonak, the hearing aid companies whose devices were included in the management programme.

## Authors' contributions

PHS and TW conceived the study and all authors contributed to the study design and data collection methods. PHS and TW performed the data collection. CG and JS performed the statistical analysis and drafting of the manuscript. All authors read and approved the final manuscript.

## Pre-publication history

The pre-publication history for this paper can be accessed here:


